# An Empirical Model-Based Algorithm for Removing Motion-Caused Artifacts in Motor Imagery EEG Data for Classification Using an Optimized CNN Model

**DOI:** 10.3390/s24237690

**Published:** 2024-11-30

**Authors:** Rajesh Kannan Megalingam, Kariparambil Sudheesh Sankardas, Sakthiprasad Kuttankulangara Manoharan

**Affiliations:** Humanitarian Technology (HuT) Labs, Department of Electronics and Communication Engineering, Amrita Vishwa Vidyapeetham, Amritapuri 690525, India; kssankardas2000@gmail.com (K.S.S.); sakthiprasadkm@am.amrita.edu (S.K.M.)

**Keywords:** brain–computer interface (BCI), motor imagery-electroencephalography (MI-EEG), motion artifacts, convolutional neural network (CNN), quadriplegics, empirical error model, wheelchair

## Abstract

Electroencephalography (EEG) is a non-invasive technique with high temporal resolution and cost-effective, portable, and easy-to-use features. Motor imagery EEG (MI-EEG) data classification is one of the key applications within brain–computer interface (BCI) systems, utilizing EEG signals from motor imagery tasks. BCI is very useful for people with severe mobility issues like quadriplegics, spinal cord injury patients, stroke patients, etc., giving them the freedom to a certain extent to perform activities without the need for a caretaker, like driving a wheelchair. However, motion artifacts can significantly affect the quality of EEG recordings. The conventional EEG enhancement algorithms are effective in removing ocular and muscle artifacts for a stationary subject but not as effective when the subject is in motion, e.g., a wheelchair user. In this research study, we propose an empirical error model-based artifact removal approach for the cross-subject classification of motor imagery (MI) EEG data using a modified CNN-based deep learning algorithm, designed to assist wheelchair users with severe mobility issues. The classification method applies to real tasks with measured EEG data, focusing on accurately interpreting motor imagery signals for practical application. The empirical error model evolved from the inertial sensor-based acceleration data of the subject in motion, the weight of the wheelchair, the weight of the subject, and the surface friction of the terrain under the wheelchair. Three different wheelchairs and five different terrains, including road, brick, concrete, carpet, and marble, are used for artifact data recording. After evaluating and benchmarking the proposed CNN and empirical model, the classification accuracy achieved is 94.04% for distinguishing between four specific classes: left, right, front, and back. This accuracy demonstrates the model’s effectiveness compared to other state-of-the-art techniques. The comparative results show that the proposed approach is a potentially effective way to raise the decoding efficiency of motor imagery BCI.

## 1. Introduction

Electroencephalography (EEG) is useful for measuring the neurophysiological activities of the human brain in clinical studies, diagnosis, and neurological investigation. With its outstanding features like zero clinical risk, portability, low cost, and non-intrusiveness, EEG is widely used in neuroscience research [1], brain–computer interface (BCI) applications [2], neurobiological and neurotransmission dysfunction, neurorehabilitation, cognitive science, etc. Motor imagery EEG (MI-EEG) data provides a type of EEG signal that is recorded from electrodes placed on the scalp while a person imagines or attempts to perform a specific movement, such as moving a hand or foot. EEG data can be used in BCI applications, allowing users to control a device or computer through their brain signals. There are 250,000 to 500,000 patients who suffer from spinal cord injury (SCI) every year globally [3]. There are over 12.2 million new strokes each year all over the world (WSO, 2022). Many of the SCI and stroke patients have moderate to severe mobility issues. A BCI-based wheelchair can be a boon to such patients whose dependence on others for mobility could be significantly reduced.

In a BCI application that uses MI-EEG data, the user first undergoes a calibration session, during which they imagine or attempt to perform specific movements while their brain signals are recorded [4]. These recordings are used to train a machine learning algorithm to recognize the user’s intention to perform different movements based on their MI-EEG data. Once the algorithm is trained, the user can use their MI-EEG signals to control a device or computer in real time. For example, they may imagine moving their left hand to move a cursor to the left on a computer screen or imagine moving their right hand to click a button. The BCI system uses the user’s MI-EEG data to detect their intention and translates it into a control signal for the device or computer. The device or computer, in most cases, is stationary. But if the device is a wheelchair for people with mobility issues, then the motion artifacts make it difficult to harvest the control information from MI-EEG signals. 

Motor imagery EEG focuses on measuring the neural activity associated with the mental simulation of movement or action. The data contain artifacts that are caused due to eye movements, head movements, muscle contractions, and electrical noises from other equipment in the environment [5]. Movement artifacts can cause shifts in the frequency spectrum of the EEG signal, leading to incorrect frequency band analysis and interpretation. The research on the removal of motion-based artifacts from EEG signals began years ago by many innovators and researchers [6,7,8]. Extracting the consequential data from the EEG signals affected by motion artifacts is a major issue. The data experts focused on noise removal and wanted efficient filters that remove the noise but keep the useful information intact [9]. ICA and BSS are common techniques for separating brain from non-brain signals in EEG data, which are especially useful in movement studies [10]. ICA is widely used for artifact removal, while multiwavelet transforms [11], like GHM with thresholding, effectively decompose and filter noisy EEG features. Additionally, presenting metrics for alternative classification models, including Support Vector Machines (SVM), would provide a more comprehensive evaluation [12]. The use of bandpass filters, notch filters [13], etc., which split the incoming data into different frequency ranges, has been explored. But such filters are not effective in removing the motion artifacts. The dry electrodes used in EEG devices are very sensitive to movement artifacts, which can drastically affect the monitoring efficiency [14]. It has been observed that though there were improvements in removing artifacts from EEG data by applying several EEG enhancement algorithms, performance was much lower compared to the EEG data obtained for stationary users [15]. The prevalent algorithms were developed to remove artifacts caused by muscle movement and eye blinks and not specifically for motion artifacts caused by mobile users, e.g., wheelchair users. 

In this study, we aim to address these challenges by proposing an empirical model-based approach for removing motion-induced artifacts from MI-EEG data, which is then processed using a modified CNN-based deep learning algorithm. The primary goal is to enhance classification accuracy for four directional commands (left, right, front, back) in real tasks within a controlled environment where a wheelchair is in motion, making the signals more prone to noise. The classification tasks we address involve real physical tasks rather than imaginary or purely visual tasks, meaning that the user performs mental simulations of movement (motor imagery) while observing directional prompts and processing intentions to control a wheelchair. The developed empirical model computes the error based on factors such as accelerometer values, surface friction of various terrains, wheelchair weight, and subject weight. The proposed empirical model-based artifact removal from MI-EEG data, combined with a deep learning-based Layer Modified CNN (LM-CNN) algorithm, enables the classification of motor imagery commands, specifically distinguishing directional commands such as right, left, forward, and backward. To accurately compute errors in the empirical model, we evaluated three different wheelchair models with varying weights and stabilities across five distinct terrains—road, brick, concrete, carpet, and marble. These variations in wheelchair type and surface affect frictional values, wheel contact, and stability, which in turn influence the magnitude and nature of the error. This approach helps model the impact of diverse real-world conditions on error generation. The empirical model computes error based on three-dimensional accelerometer measurements (ax, ay, az), the surface friction values of the five different terrains, the weight of the three wheelchairs, and the weight of the subjects. The model works as an efficient and intelligent filter that can be used with live EEG sensor data. The filtered data is used to train the LM-CNN model to predict directional command classes—specifically, right, left, forward, and backward—for the goal of wheelchair navigation using motor imagery EEG signals. The proposed empirical model-based LM-CNN method gives an average accuracy of 94.04% compared to other state-of-the-art techniques.

The remainder of the paper is organized as follows. In Section 2, we present the state-of-the-art research work related to classification of motor imagery EEG data using image processing methods, neural networks, various ways to remove artifacts, EEG based wheelchair navigation, etc. This is followed by Section 3, in which the proposed empirical error model-based artifact removal for the classification of MI-EEG data using a modified CNN-based deep learning algorithm for wheelchair users with severe mobility issues is discussed. Section 5 presents the validation of the proposed methods and benchmarking, and is followed by Section 6.

## 2. Literature Review

There are several studies in the literature that use CNN for the classification of motor imagery data. The research study [16] explores the advantages of extracting and merging multilevel convolutional features from several CNN layers, providing more robust and detailed representations of the EEG motor imagery data. This approach enhances classification accuracy by capturing intricate patterns in the EEG signals, which is particularly beneficial for distinguishing between motor imagery commands with greater precision in BCI applications. The suggested CNN model can extract reliable spectral and temporal information from the unprocessed EEG data. However, the proposed model was only tested on one kind of MI-EEG dataset, and thus cannot be used as a generalized method. The experiments discussed in Ref. [17] demonstrate that using fewer electrodes—such as configurations with 13, 9, 6, or even as few as 3 electrodes—can still achieve the effective classification of motor imagery commands (right, left, forward, and backward), compared to the standard configurations that typically involve 64 or 128 electrodes. With fewer electrodes, the authors demonstrated that the EEG decoding method achieved an accuracy of 83.2% and a false positive rate of 19.0% in classifying motor imagery commands. Another study [18] suggested a discriminative feature learning technique that enhances the identification of distinct features for each motor imagery class, specifically targeting tasks such as left-hand and right-hand motor imagery. This helps in gaining improved classification accuracy. Shallow convolutional networks [19] and feature similarity-based weighted ensemble learning [20] focus on building an efficient CNN-based classification algorithm. All these methods consider the subject to be stationary. These methods are not suitable for mobile subjects, which generate a considerable amount of motion artifacts. 

However, some researchers classified motor imagery EEG data using methods other than CNN. The backpropagation neural network (BP-NN) [21], SessionNet [20], and Least Squares Support Vector Machine (LS-SVM) [22] are a few of the efforts made to build an efficient model to perform this classification. Even though these models have promising results, CNN-based models are more promising in the case of real-time scenarios.

EEG signals can be contaminated with various types of artifacts, such as eye blinks, muscle activity, and environmental noise. Some of the various ways to remove artifacts from EEG signals include the following: filtering, independent component analysis (ICA), regression-based techniques, wavelet transform, artifact rejection, template subtraction, adaptive filtering, and multivariate fast and adaptive empirical mode decomposition; all of these are presented in different research articles [6,7,23,24,25]. A wavelet and canonical correlation analysis (CCA) combination approach has been proposed as the pre-processing step to remove artifacts for a BCI-based ground vehicle control [26]. In essence, this method makes use of the common spatial pattern (CSP), which has the capacity to extract the feature of event-related potentials.

Without using an arbitrary threshold to detect the artefactual components [27], a hybrid method for automatically identifying and removing artefactual components in EEG signals has been proposed. The study focused on elimination of the artefactual components. with the least amount of distortion to the desired brain signals observed when wavelet multiresolution analysis (WMA) and ICA were used together. In Ref. [28], the Short Time Fourier Transform (STFT) signal pre-processing approach was applied to transform raw EEG signals into time-frequency pictures. A more reliable multi-scale feature was created by flattening the high-level features from the final convolutional layer and the low-level features from the final pooling layer. However, image-based conversion made the system slow and less promising for real-time prediction systems. 

Many methods have been explored for motor imagery EEG decoding using CNN [8,29,30,31,32]. The study [33] proposed a Channel Projection (CP)-Mixed Net technique with amplitude-perturbation data augmentation. The authors of Ref. [34] proposed a new support vector neural network (SVNN) method for classifying the EEG signal of the subjects performing motor imagery into four classes: right hand, left hand, right leg, and left leg, respectively. In another study [35], a clustering-based multitask feature learning algorithm for improved EEG pattern decoding was proposed. The novelty in this work is that the researchers explored the potential subclasses using the AP clustering algorithm to characterize the intrinsic sample structure of EEG data. The proposed algorithm was not tested with other clustering algorithms. This would have helped them increase their benchmarking value of the proposed system.

Ref. [36] employed an empirical mode decomposition (EMD) as a pre-processing technique in which the signals are decomposed to IMFs. Another empirical-based error correction model is proposed in Ref. [37], which considers the accelerometer data (AC) of subjects in motion. In the paper, they addressed the effect of motion-induced artifacts in the EEG data. A combination of EMD and Dynamic Differential Entropy (DDE) has also been proposed as a pre-processing technique for subject-independent EEG classification using CNN [7]. 

The most commonly used wheelchair control method is the joystick-based control. Other methods include switch control, head control, tongue control, sip and puff control, eye tracking control, voice control, etc. [38]. These kinds of control methods for a wheelchair cannot be used by patients who are quadriplegic or have diplegia. An IR sensor-based hand gesture-controlled wheelchair is proposed in Ref. [38], which was designed for people with mobility issues like stroke patients and spinal cord injury patients. But this can be used only if a certain minimum level of motor function is recovered in their hands. Autonomous wheelchairs that are based on LiDAR are presented in Refs. [39,40,41,42]. These wheelchairs are expensive and have only been implemented indoors. In addition, these autonomous wheelchairs are still not widely used, even by people with minimal mobility issues. 

EEG-based powered wheelchairs with improved dry electrodes are becoming more popular in the current technological era mainly due to their vast field of application [43,44]. The subjects in wheelchairs undergo sudden motions and jerking depending upon the terrain they move on. The head of the subject is always in perpetual motion until the wheelchair stops. Thus, implementing a BCI-controlled wheelchair becomes a tricky maneuver. A few of the efforts made to control a powered wheelchair using MI-EEG data are presented in Refs. [45,46,47,48]. Even though an accuracy of 85% is achieved on average when using EEG data to control wheelchairs, the effect of motion artifacts caused due to wheelchair movement have not been addressed. Even the hair on the scalp can create noise. The research study [49] addressed the issue of using wet electrodes and proposed a noncontact dry electrode with an adaptive mechanical design. This method faced issues when the thickness and humidity of the hair layer varied. The main contributions of this work are the following:

- Modified the CNN model for motor imagery EEG data classification of subjects in motion.
- The empirical error model with four main parameters that identify motion artifacts.
- The experimental setup and the MI-EEG dataset of five mobile subjects recorded in five different terrains with three different teleoperated electric wheelchairs. In each condition, three trials were carried out, providing a total of 225 EEG recordings.
- Evaluation and benchmarking of the proposed method with current state-of-the-art methods.

## 3. Materials and Methods

### 3.1. System Architecture

**Figure 1:**
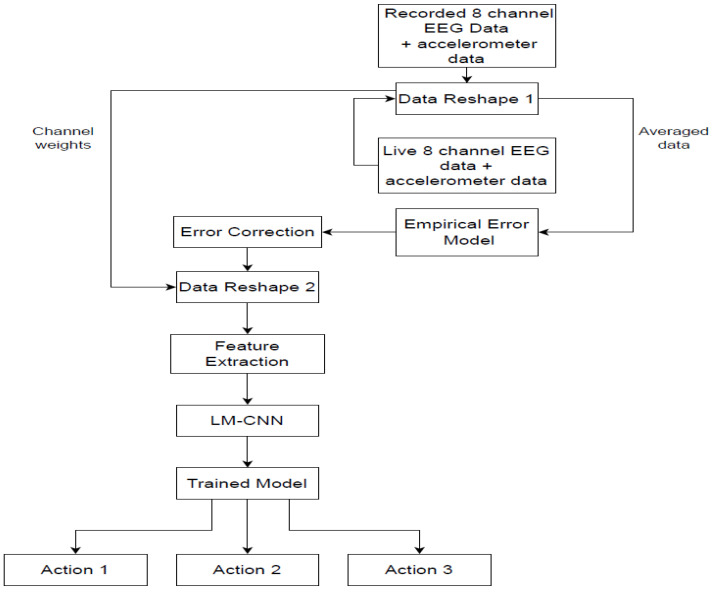
Overall architecture of the proposed empirical model-based artifacts removal and LM-CNN-based classification.

As shown in Figure 1, the recorded 8-channel MI-EEG and accelerometer data undergo initial data preprocessing and reshaping steps to reduce noise and prepare the data for the empirical error model. Two data reshaping techniques are used to make the data usable for the empirical error model. Data Reshape 1 involves averaging (*E_avg*) the EEG data across all eight channels to produce a single-channel value. The reshaped data from Data Reshape 1 are then inputted into the empirical error model for further processing. The empirical model computes the ‘error’ by measuring the deviation between reference accelerometer values—collected in a standard, comfortable environment—and values collected during active motion. This error accounts for variations due to accelerometer readings across three axes, surface friction on five different terrains, the weights of the five subjects, and the weights of the three wheelchairs. Once the error model has the required inputs, the empirical equations are computed. This error model helps adjust the EEG data across different terrains and wheelchair designs, thereby improving accuracy and decision-making in real-world conditions. Expanding the model’s detail on parameters like weight, surface friction, and their integration will facilitate better understanding and replication of this approach in diverse settings. Following this, error correction is applied to adjust for any remaining discrepancies identified by the empirical model, enhancing the data quality before input into the LM-CNN. Next, Data Reshape 2 restores the single-channel output back to an 8-channel format using the previously calculated channel weights. The labeled EEG data, corresponding to specific motor imagery actions (categorized as Actions 1, 2, and 3), is used in the feature extraction process during each recording session. Signal-based, time-based, and frequency-based features are extracted and given to the LM-CNN for training.

**Figure 2:**
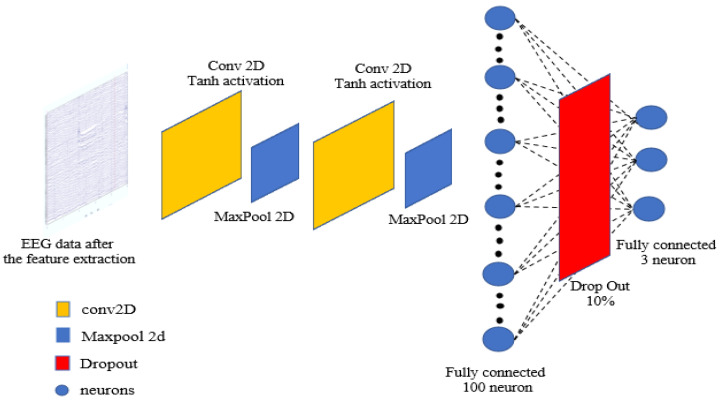
The proposed LM-CNN architecture.

The feature extraction process produces 16 different features. The neural networks are trained with these generated features. Once trained, whenever the live EEG data is provided as input to the model after reshaping, the empirical error (the difference between reference data and motion data) is calculated. The trained LM-CNN model then predicts the class label, corresponding to specific motor imagery commands such as ‘right’, ‘left’, ‘forward’, or ‘backward’. The empirical model proposed works as an efficient and intelligent filter that can be used with live EEG sensor data, which refers to the motor imagery EEG data recorded in real-time while a person is sitting and operating the wheelchair. Figure 2 shows the details of the proposed LM-CNN model. The model is a layer-modified CNN with two convolutional 2D layers, one max-pooling-2D layer, and one dropout layer. The batch size and the number of epochs is selected using the grid search CV optimization method. To manage the inherent variability in EEG data, batch normalization is strategically implemented after each convolutional layer, stabilizing and accelerating the training process. The network transitions to fully connected layers through a flattening operation, followed by a dense layer of 100 units with ReLU activation and a dropout rate of 0.5 to prevent overfitting. The final output layer consists of 3 units with softmax activation for three-class classification. The model is optimized using the Adam optimizer with a learning rate of 0.001, beta_1 of 0.9, and beta_2 of 0.999, chosen specifically for its adaptive learning capabilities in handling EEG data characteristics.

### 3.2. Data Collection

**Figure 3:**
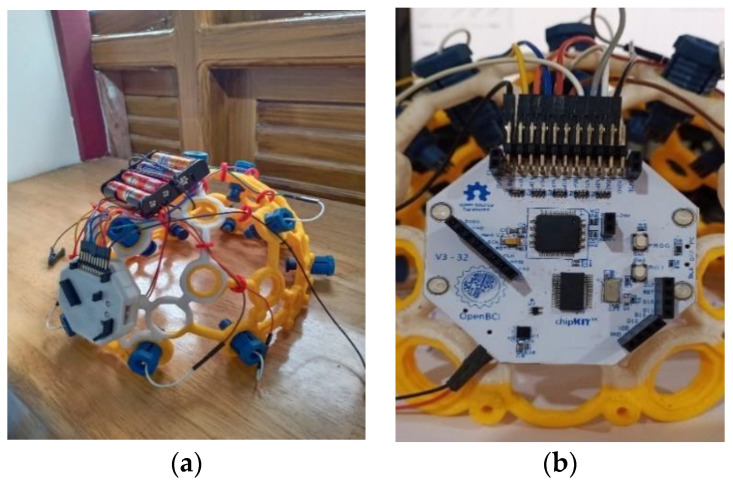
(**a**) Image of the ultra-cortex ‘Mark IV’; (**b**) Cyton Board.

Motor imagery EEG data were recorded using the 8-channel OpenBCI Cyton board 230 (Brooklyn, NY, USA), which is shown in Figure 3. The OpenBCI Cyton board plays a crucial role in data collection by serving as the primary interface for capturing and digitizing EEG signals. Key components of the Cyton board include the ADS1299 Analog Front End, which is responsible for high-resolution EEG signal acquisition, and the PIC32MX250F128B microcontroller, which processes the data. The board also features an RFduino BLE radio for wireless data transmission and an LIS3DH 3-axis accelerometer for motion detection. The Cyton board operates in conjunction with the OpenBCI GUI software (Version V5.0.0), which facilitates real-time data visualization and recording. This software allows users to configure the board, monitor signal quality, and save data for further analysis. The integration of the Cyton board with the OpenBCI GUI ensures a seamless workflow for brain–computer interface (BCI) applications, enabling efficient and accurate data collection. Each time a motor action is shown on the screen, the user must mentally perform the action without any overt movement or without any peripheral (muscle) activation. During each recording session, the subject is given two motor imagery tasks to perform—right and left-hand movements, each for 5 s. The recording session involves the following steps:

- Once the configuration is complete, a visual cue appears on the right side of the screen. Each of the visual cues is shown for 5 s.
- The first cue is RELAX. Wherever this word appears on the right side of the screen, the subject needs to relax.
- The next cue can be either of the two words LEFT or RIGHT. The words are randomly chosen and displayed.
- If the cue is LEFT, the subject must imagine moving his/her left hand. The subject imagines lifting the left hand up and down until the cue disappears.
- A RELAX cue appears after the LEFT cue disappears. Then the subject relaxes.
- If the cue is RIGHT, the subject must imagine moving his/her right hand. The subject imagines lifting the right hand up and down until the cue disappears.
- A RELAX cue appears after the RIGHT cue disappears. Then the subject relaxes.

**Figure 4:**
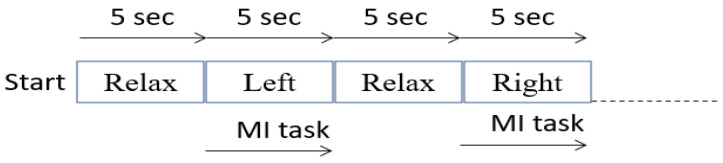
Order and duration of motor actions during a recording session.

Figure 4 shows the order of motor action. The incoming data during each recording are labelled and saved. Data were collected from five healthy subjects. All five subjects were males and did not have any recent serious medical issues. EEG data recordings were carried out in two different experimental setups. The first was the reference EEG data recording of a subject in an artifact-free setup, and the second was the EEG data recording of the subject in motion, which are explained in the upcoming sections. The reference EEG data are collected specifically for error calculations and empirical modeling. This dataset is not included in the main dataset used for training or other purposes. A total of seventy-five recordings are captured using the second experimental setup for the five subjects, which formed the dataset for our proposed work.

### 3.3. EEG Data Recording Reference 

**Figure 5:**
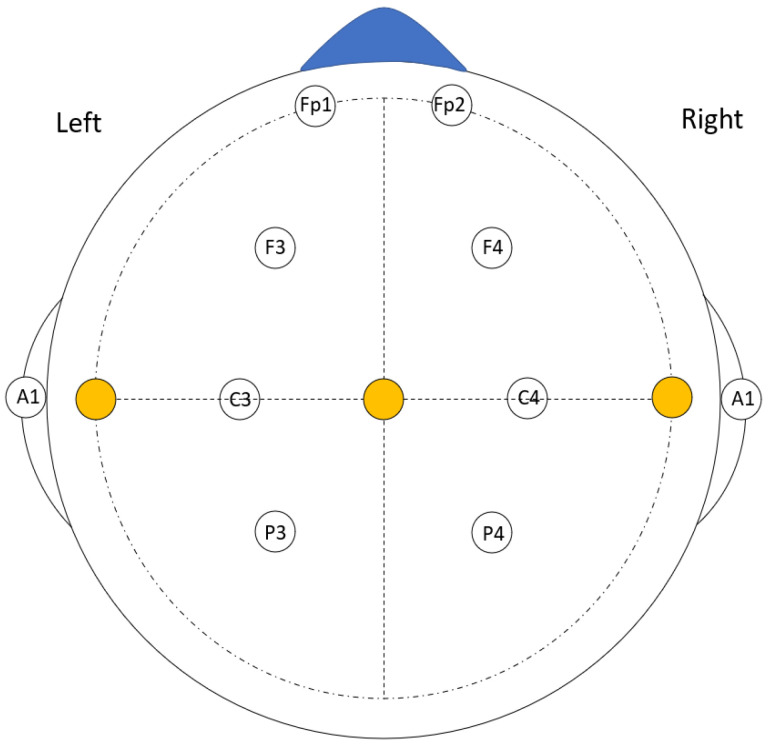
The electrode positions used during the recordings.

**Figure 6:**
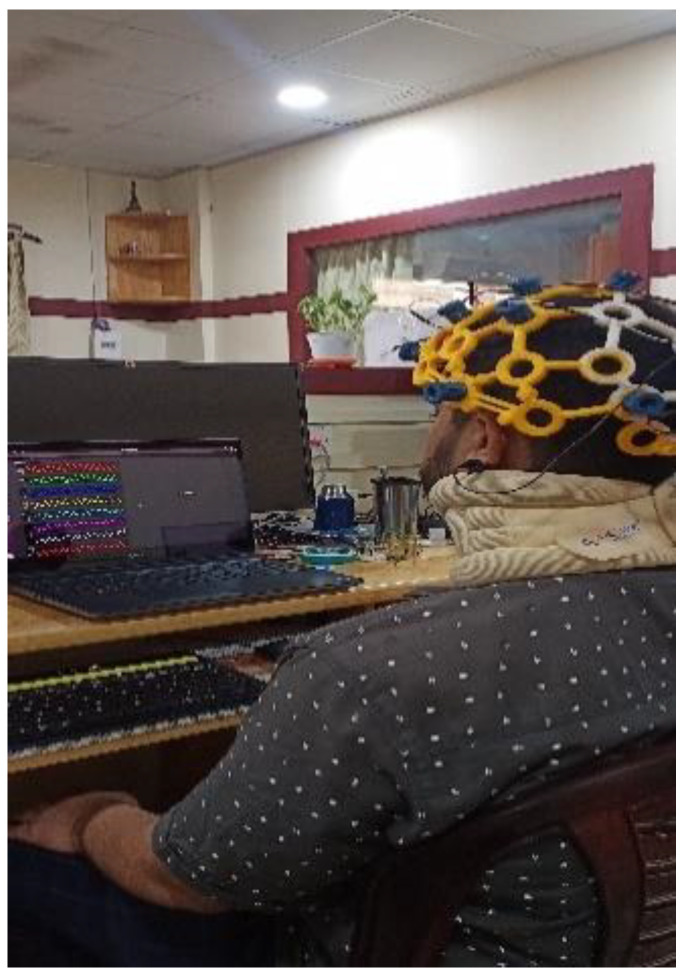
The image shows a sample reference EEG recording session.

For this data recording session, the subject was seated at a computer table with a laptop placed 50–60 cm away in a well-lit room without any noise disturbances. Each subject wore a neck band to reduce the artifacts caused by any jerk or head movement. They were also instructed not to make sudden body, eye, and head movements to minimize the effect of electromyography (EMG) signals from the data. The EEG data, along with the accelerometer data, were recorded during this recording. These data were the main reference data with which the empirical model calculated the error. The EEG electrodes were placed in Fp1, Fp2, F3, F4, C3, C4, P3, and P4 as shown in Figure 5. These positions were maintained in all the recording sessions throughout the experiment for each of the subjects. Figure 6 shows the experimental setup for reference data recording.

## 4. Empirical Modeling 

**Figure 7:**
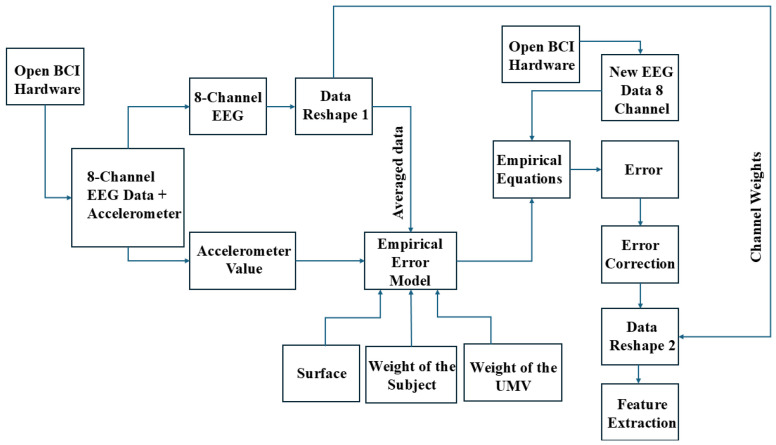
Empirical error model creation and the flow chart.

The empirical model is formulated based on the reference EEG data and accelerometer data. Figure 7 shows the proposed empirical error model architecture in detail. The subject wears a cap equipped with the OpenBCI Cyton board. EEG electrodes attached to the cap capture brain signals and transmitted them to the Cyton board. The board digitizes these signals and sends the data via USB to a connected laptop. The OpenBCI GUI software on the laptop facilitates real-time data visualization and initial processing. After this, the data are passed to a Python script for further processing and error modeling. All data, including EEG recordings and accelerometer measurements, are transmitted to a laptop via USB. The laptop serves as a temporary storage location, holding the data only briefly for real-time processing. The system does not retain data long-term, focusing instead on immediate analysis and real-time processing using machine learning algorithms without permanent data storage. Finally, the processed data are fed into a Convolutional Neural Network (CNN) for analysis. The ‘error’ in this context refers to the difference between the baseline reference data (collected in a controlled, standard environment) and the live data recorded while the wheelchair is in motion across different terrains. The empirical model can be divided into the accelerometer error model and the 8-channel EEG error model. Both these models use the same reference EEG data and the EEG motion data, which is explained in Section 5. In the Data Reshape 1 block, the 8-channel EEG data are converted to a single channel value by averaging the (*E_avg*) data from all channels. In addition to averaging, the weights of all channels are calculated. These single channel data go to the empirical model for noise removal. The Data Reshape 2 block converts the empirical model output (filtered data) into 8-channel data using the weights obtained in the Data Reshape 1 block.

### 4.1. Accelerometer Error Model

The error in the accelerometer is due to three factors: the variation in the frictional coefficient of the surface, weight of the subject, and the weight of the wheelchair. The error model is formulated based on these three factors.

#### 4.1.1. Surface

In this study, the error calculated on surface s1 (*E_axs1*) is used as the reference because it represents a stable, high-friction, and low-vibration environment, which minimizes artifacts. This surface ensures optimal contact without slippage, providing a reliable baseline for relative error comparisons across different surfaces (s2–s5). Using a reference measurement from a stable surface is supported in the literature on relative EEG measurements (e.g., [50]) to enhance the consistency and comparability of signal quality across conditions. By keeping the weight of the subject and wheelchair constant at 85 Kg and 80 Kg, respectively, the error in the accelerometer data is calculated. The accelerometer error equations in x axis for s2 to s5 are represented by Equations (1)–(4).

(1) $$E_{axs2}=\frac{{µ}_{s1}}{{µ}_{s2}}\;{\times \;Wf}_{axs1}\times E_{axs1}$$

(2) $$E_{axs3}=\frac{{µ}_{s1}\;}{{µ}_{s3}\;}\;{\times \;Wf}_{axs2}\times E_{axs1}$$

(3) $$E_{axs4}=\frac{{µ}_{s1}\;}{{µ}_{s4}\;}\;{\times \;Wf}_{axs3}\times E_{axs1}$$

(4) $$E_{axs5}=\frac{{µ}_{s1}\;}{{µ}_{s5}\;}\;{\times \;Wf}_{axs4}\times E_{axs1}$$

The terms ${Wf}_{axs1},\;{Wf}_{axs2},\;{Wf}_{axs3},\;and\;{Wf}_{axs4}$ are the weight factors that were computed from each case, and the average of all four weight factors is represented as ${Wf}_{axsn}$. Using the four equations, the general equation to compute the error caused in the accelerometer data (without correction factor) due to a surface is computed using Equation (5):

(5) $$E_{axsn}=\frac{{µ}_{1}\;}{{µ}_{n}\;}\;\;{\times \;Wf}_{axsn}\;\times E_{axs1}$$

Two-level error correction $\left(E_{axs4e}-E_{axs4}\right)$ and $\left(E_{axs5e}-E_{axs5}\right)$ are calculated to find the correction factor (Cf). Equation (6) (${Cf}_{axs}$) is the correction factor with respect to the surface and error in the x axis.

(6) $${Cf}_{axs}=\frac{\left(E_{axs4e}-E_{axs4}\right)+(E_{axs5e}-E_{axs5})}{2}$$

(7) $$E_{axsn}=\frac{{µ}_{s1}\;}{{µ}_{sn}\;}\;\;{\times \;Wf}_{axsn}\times E_{axs1}+{Cf}_{axs}$$

Similar to the general equation for error in the x axis of Equation (7) ($E_{axsn}$), Equations (8) and (9) are the general equations for the y and z axes. Table 1 shows the symbols used in this section.

(8) $$E_{aysn}=\frac{{µ}_{s1}\;}{{µ}_{sn}\;}\;\;{\times \;Wf}_{aysn}\times E_{ays1}+{Cf}_{ays}$$

(9) $$E_{azsn}=\frac{{µ}_{s1}\;}{{µ}_{sn}\;}\;\;{\times \;Wf}_{azsn}\times E_{azs1}+{Cf}_{azs}$$

#### 4.1.2. Subject Weight

Next, we find the error considering the weight of each subject. The first subject’s weight ($W_{1}$) and the error calculated from it, $E_{axp1}$, are taken as reference. By keeping the frictional coefficient of the surface and weight of the wheelchair constant, the errors in accelerometer data for the three axes (Ax, Ay, Az) for four subject weights ($W_{2}$–$W_{5}$) are calculated. Equations (10)–(12) are the general equations for Ax, Ay, and Az, in which ${CF}_{axp}$, ${CF}_{ayp},\;{and\;CF}_{azp}$ are the correction factors for the x, y, and z axes. Table 2 shows the symbols used in this section.

(10) $$E_{axpn}=\frac{W_{1}\;}{W_{n}\;}\;\;{\times \;Wf}_{axpn}\times E_{axp1}+{Cf}_{axp}$$

(11) $$E_{aypn}=\frac{W_{1}\;}{W_{n}\;}\;\times {\;Wf}_{aypn}\times E_{ayp1}+{Cf}_{ayp}$$

(12) $$E_{azpn}=\frac{W_{1}\;}{W_{n}\;}\;\;{\times \;Wf}_{azpn}\times E_{azp1}+{Cf}_{azp}$$

#### 4.1.3. Wheelchair Weight

Finally, we find the error considering the weight of each wheelchair. The first wheelchair weight ($U_{1}$) and the error calculated from it, $E_{axw1}$, are taken as reference. By keeping the frictional coefficient of the surface and the subject weight constant, the error in accelerometer data for the three axes (Ax, Ay, Az) for the remaining two wheelchair weights ($U_{2}$ and $U_{3}$) is calculated. Equations (13)–(15) are the general equations for Ax, Ay, and Az, in which ${Cf}_{axw},$ ${Cf}_{ayw}$, and ${Cf}_{azw}$ are the correction factors for the x, y, and z axes. Table 3 shows the symbols used in this section.

(13) $$E_{axwn}=\frac{U_{1}\;}{U_{n}\;}\;\;{\times \;Wf}_{axwn}\times E_{axw1}+{Cf}_{axw}$$

(14) $$E_{aywn}=\frac{U_{1}\;}{U_{n}\;}\;\;{\times \;Wf}_{aywn}\times E_{ayw1}+{Cf}_{ayw}$$

(15) $$E_{azwn}=\frac{U_{1}\;}{U_{n}\;}\;\;{\times \;Wf}_{azwn}\times E_{azw1}+{Cf}_{azw}$$

We thereby obtain the individual error equations for each factor and each accelerometer axis. The combined error equations for all the three axes are given as follows:

(16) $$E_{asn}=\sqrt{{{(E}_{axsn})}^{2}+{{(E}_{aysn})}^{2}+{{(E}_{azsn})}^{2}}$$

(17) $$E_{apn}=\sqrt{{{(E}_{axsn})}^{2}+{{(E}_{aysn})}^{2}+{{(E}_{azsn})}^{2}}$$

(18) $$E_{awn}=\sqrt{{{(E}_{axwn})}^{2}+{{(E}_{aywn})}^{2}+{{(E}_{azwn})}^{2}}$$

The error in accelerometer data in (19), $E_{a}$, is the average of the error in all three factors that are found using Equations (16)–(18).

(19) $$E_{a}=\frac{E_{asn}+E_{apn}+E_{awn}}{3}$$

### 4.2. 8–Channel EEG Error Model

The next step is to calculate the error in the EEG data that is recorded simultaneously with the accelerometer data. We compute the error for the same three factors that we considered during the accelerometer error calculation. E_s1 is the error for the first surface (s1) and is taken as reference, and the error in the remaining four surfaces (s2–s5) are found using Equations (20) to (23), which are given below.

(20) $$E_{s2}=\frac{{µ}_{1}\;}{{µ}_{2}\;}\;{\times \;Wf}_{s1}\times E_{s1}\;$$

(21) $$E_{s3}=\frac{{µ}_{1}\;}{{µ}_{3}\;}\;{\times \;Wf}_{s2}\times E_{s1}$$

(22) $$E_{s4}=\frac{{µ}_{1}\;}{{µ}_{4}\;}\;{\times \;Wf}_{s3}\times E_{s1}$$

(23) $$E_{s5}=\frac{{µ}_{1}\;}{{µ}_{5}\;}\;{\times \;Wf}_{s4}\times E_{s1}$$

The general equation to find error (without the correction factor) on another surface is given below.

(24) $$E_{sn}=\frac{{µ}_{1}\;}{{µ}_{n}\;}\;\;{\times \;Wf}_{sn}\times E_{s1}$$

Two-level error correction $\left(E_{s4e}-E_{s4}\right)$ and $\left(E_{s5e}-E_{s5}\right)$ are calculated to find the correction factor (Cf). ${Cf}_{s}$ is the correction factor with respect to the surface.

(25) $${Cf}_{s}=\frac{\left(E_{s4e}-E_{s4}\right)+(E_{s5e}-E_{s5})}{2}$$

$E_{sn}$ is the general equation that is found after including the Cf.

(26) $$E_{sn}=\frac{{µ}_{1}\;}{{µ}_{n}\;}\;\;{\times \;Wf}_{sn}\times E_{s1}+{Cf}_{s}$$

Similarly, the general equations for error considering the weight of the subject ($E_{pn}$) and the weight of the wheelchair ($E_{wn}$) can be formulated as follows:

(27) $$E_{pn}=\frac{W_{1}\;}{W_{n}\;}\;\;{\times \;Wf}_{pn}\times E_{p1}+{Cf}_{p}$$

(28) $$E_{wn}=\frac{U_{1}\;}{U_{n}\;}\;\;{\times \;Wf}_{wn}\times E_{w1}+{Cf}_{w}\;$$

The overall error contributed by the three factors, including surface coefficient, subject weight, and wheelchair weight, is the average of $E_{sn},\;E_{pn}$, $E_{wn}$, and $E_{a}$.

(29) $$E=\frac{{(E}_{sn}+E_{pn}+E_{wn}+E_{a})}{4}$$

### 4.3. Feature Extraction

In addition to the empirical error model, the feature extraction process is carried out, which helps in making the data more discrete for each class and thus increases the classification accuracy. The 16 features are extracted collectively from all eight EEG channels at specific data points rather than from each channel individually. This extraction is event-based, not tied to a time window, task, or trial. Mobility, complexity, kurtosis, mean, max, coefficient of variation, skewness, wavelet detailed energy, wavelet detailed entropy, variance, FFT delta max power, FFT theta max power, FFT alpha max power, and FFT beta max power are the key features extracted from the EEG data. Delta, theta, alpha, and beta are the major frequency level features which are used in clinical analysis. The strength of these bands in a person’s EEG data are also related to his/her emotional and mental state. Kurtosis, mean, and variance are standard statistical tools normally used in classification algorithms. Although feature extraction is typically part of CNN modeling, our dataset shows high variability, making it challenging to achieve accurate results with direct data input. By performing explicit feature extraction, we provide a more stable, representative dataset that improves classification accuracy. After feature extraction, there are 10,000 data samples for each class and 30,000 in total. This is the final training data that are given to the proposed CNN model. Here, “samples” refer to instances of data taken at 5-s intervals, with each sample containing feature information derived from the EEG and accelerometer data.

## 5. Experiments and Results

### 5.1. EEG Data Recording in Motion

**Figure 8:**
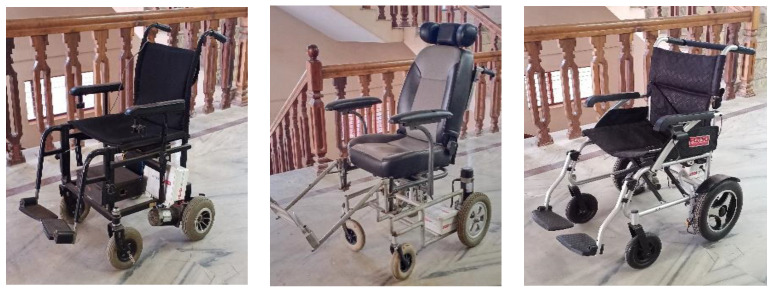
The three different wheelchairs that were built for the EEG data recording purpose.

For recording EEG data in motion, three different powered wheelchairs are designed and implemented, as shown in Figure 8, which are powered by Raspberry Pi 3 (Raspberry Pi Ltd., Cambridge, UK). One of the three wheelchairs has a 350 W, 24 V, geared PMDC motor with rubber tires and weighs 90 Kg. The remaining two have 250 W, 24 V geared DC motors with similar rubber tires and weigh 80 Kg and 25 Kg, respectively. As mentioned earlier, five subjects took part in this data recording session. The wheelchairs are teleoperated using a Bluetooth joystick with the same speed of 6 km/h. Table 4 provides the details of the subjects, including their age and weights. Table 5 lists different terrains and their friction coefficients.

**Figure 9:**
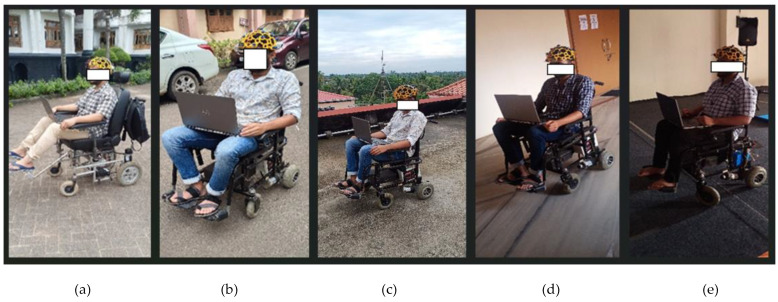
The above images show the recording sessions we performed in the artifact EEG recording: (**a**) wheelchair-2 on concrete surface, (**b**) wheelchair-3 on a brick surface, (**c**) wheelchair-2 on the road, (**d**) wheelchair-2 on the marble surface, (**e**) wheelchair-2 on the carpet surface.

During this data recording session, the five subjects sat on three different wheelchairs and navigated over five different terrains with different surface friction coefficients for each terrain. The five different terrains were a marble floor, concrete floor, carpeted floor, interlock brick floor, and road. Each subject received the same number of motor actions to imagine in each recording session. A few images that were taken during the recording sessions are shown in Figure 9. The main motive behind this recording setup was to find the effect and changes that happen to the EEG data when the subject is in the same wheelchair but on different terrains, and on the same terrain but using different wheelchairs. This helps in creating a real-time dataset with artifacts, which is useful to validate the empirical model.

### 5.2. Empirical Error Model Validation

To validate the empirical error model, we measure performance metrics such as accuracy, precision, recall, and F1-score, before and after the application of the error model, for four different cases:

1. The classification of the EEG data measured without applying the empirical error model for a subject with a single wheelchair and five different surfaces. EEG data are allocated to each class according to the motor imagery task performed during recording, and data are labeled based on these tasks. The dataset is divided into training and testing sets, with an 80:20 split to validate model performance.
2. The classification of the EEG data measured without applying the empirical error model for a subject with three different wheelchairs and single surface.
3. The classification of the EEG data measured by applying the empirical error model for a subject with a single wheelchair and five different surfaces.
4. The classification of the EEG data measured by applying the empirical error model for a subject with three different wheelchairs and a single surface.

**Figure 10:**
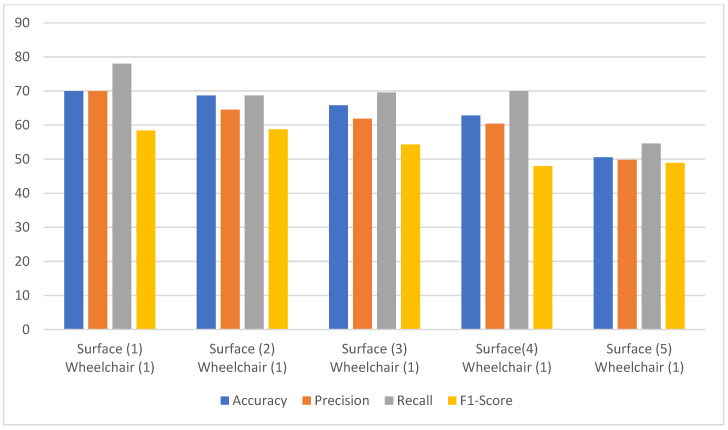
The model performance comparison for a subject in the same wheelchair but on different surfaces without the empirical model.

**Figure 11:**
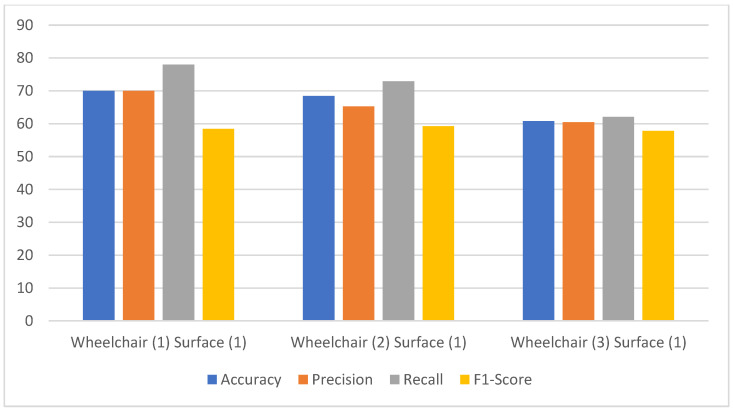
The model performance comparison for a subject on the same surface but using different wheelchairs without the empirical model.

Figure 10 shows the performance metrics comparison for a subject in the same wheelchair but on different surfaces, without the empirical model as in case (a). The accuracy variation is about 20%. The highest accuracy is only 70%, and the lowest is 50.57%. This variation in the accuracy also shows the effect of noise concerning changes in the surface. The lowest accuracy was obtained for surface 5, with the highest surface friction value. Similarly, the variation in precision and recall is also observed to be about 20% for all five surfaces, whereas it is about 10% for the F1-score. But the F1-score had the lowest range of all four metrics, only ranging from 48–58%. Figure 11 shows the performance metrics without the use of the empirical model for case (b). In this case, the accuracy and precision also ranged from 60% to 70%, whereas only the recall could reach 78% for wheelchair 1. The recall could still not cross the 80% mark. The F1-score remained almost constant at 58%, which is the lowest of all the metrics.

**Figure 12:**
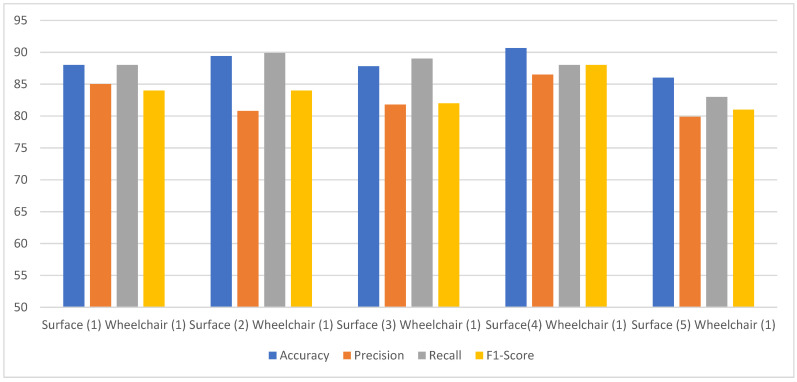
The proposed model performance comparison of a subject in the same wheelchair but on different surfaces.

**Figure 13:**
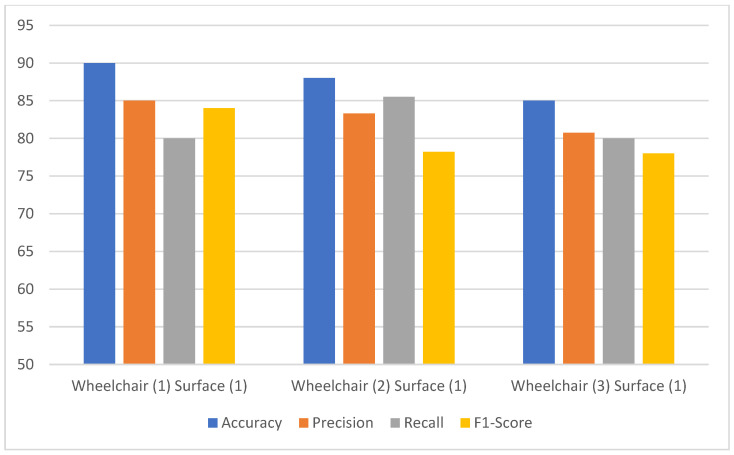
The proposed model performance comparison of a subject in the same wheelchair but on different surfaces.

Figure 12 shows the performance comparison of the proposed model on EEG data from a subject using the same wheelchair across different surfaces, with and without applying the empirical error model, as in case (c). The empirical error model is applied before the classification step, helping to standardize and adjust data variability due to surface differences. The empirical error model is applied before the classification step. This step standardizes and adjusts for data variability resulting from surface differences, enhancing the consistency of data input to the classifier. It is clearly observed that the accuracy could reach 90%, ranging from 86 to 90% for the various surfaces. The other parameters—precision, recall, and F1-scores for various surfaces—only varied within a 7–8% range. The range for precision was 81–87%, 83–90% for recall, and 81–88% for the F1-score, all well ahead of the metrics for case (a). We observed a maximum increase of 20% in accuracy, 18% in precision, 12% in recall and 20% in F1-score when compared to that of the scenario in case (b). Thus, the proposed empirical model is very effective in removing the motion noise artifacts from the EEG data. This can be further confirmed from the test results for case (d), as shown in Figure 13. An accuracy of 90% in wheelchair 1, 88% in wheelchair 2, and 85% in wheelchair 3 was achieved. On the other hand, for case (b), the accuracies for wheelchairs 1, 2, and 3 were 70%, 69%, and 80% respectively, which again shows that the maximum increase in accuracy is 20%. The other parameters—precision, recall, and F1-score—also showed a significant increase in the performance metrics. 

The proposed model was compared with the state-of-the-art techniques for LM-CNN model evaluation, empirical model evaluation, and overall system evaluation. In all three stages, the evaluation was based on the classification report of the classifier. The classification accuracy is given the highest priority, and precision, recall, and F1-score are the other performance metrics used for benchmarking. 

### 5.3. LM-CNN Model Evaluation

The performance of the proposed CNN model is compared with three other CNN models presented in various previous studies. Our proposed CNN model showed better results when compared with the other three models.

In the case of precision, CNN4 performed better than ours. CNN4 has a precision of 74.32%, which is around 10% higher than our proposed model. But, in the case of recall and F1-score, our model obtained higher values. The proposed LM-CNN model has an 80.23% accuracy, being the highest among all four models. Table 6 and Table 7 present the details and the testing accuracy of each model that is evaluated. In the LM-CNN model, Tanh is the activation function used in the convolutional 2D layer, whereas it is ReLu in all the other models.

### 5.4. Empirical Model Evaluation

In the proposed method, the data processing involves the empirical error model prior to the feature extraction. The model to be compared with this was another empirical error model [37], which considers only the accelerometer data (AC) as a parameter for error correction. The second model [36] that was compared used an empirical mode decomposition (EMD) as a pre-processing technique, and the third model employed a discrete wavelet transform (DWT) [53] as a pre-processing method before providing the data for training. Out of all these three models, LM-CNN combined with the proposed empirical model (EM) and feature extraction showed the best classification accuracy of 94.40%. The proposed model also holds the highest value among all the other performance metrics. Table 8 shows the comparative results of each model with our proposed model. The results reported in Table 8 correspond to an evaluation of the empirical error model applied in the scenario of a single wheelchair on a single surface.

### 5.5. Overall System Evaluation

The performance of the proposed system, which uses empirical model-based artifact removal in motor imagery EEG data and classification using CNN, is compared with two other systems using different CNN models. The first system is a combination of the empirical model with only accelerometer values and a 7-layer CNN model [30]. The second system is composed of EMD combined with another 7-layer CNN [36]. The proposed system is a combination of accelerometer, subject weight, surface friction coefficient, and wheelchair weight with a 7-layer CNN, which outperformed the other two systems. The proposed model has an accuracy of 94.40%, a precision of 94.42%, a recall of 96.2%, and an F1-score of 90.4%, which are the highest values compared with the other two models. The 94% accuracy reported reflects correct classification at the sample level. The evaluation checks if the model correctly classifies each individual sample based on the intended task (e.g., classifying “left” when “left” is applied). This shows that the LM-CNN is efficacious when compared with other CNN models. Table 9 contains the performance results of the proposed model and the other two models.

**Figure 14:**
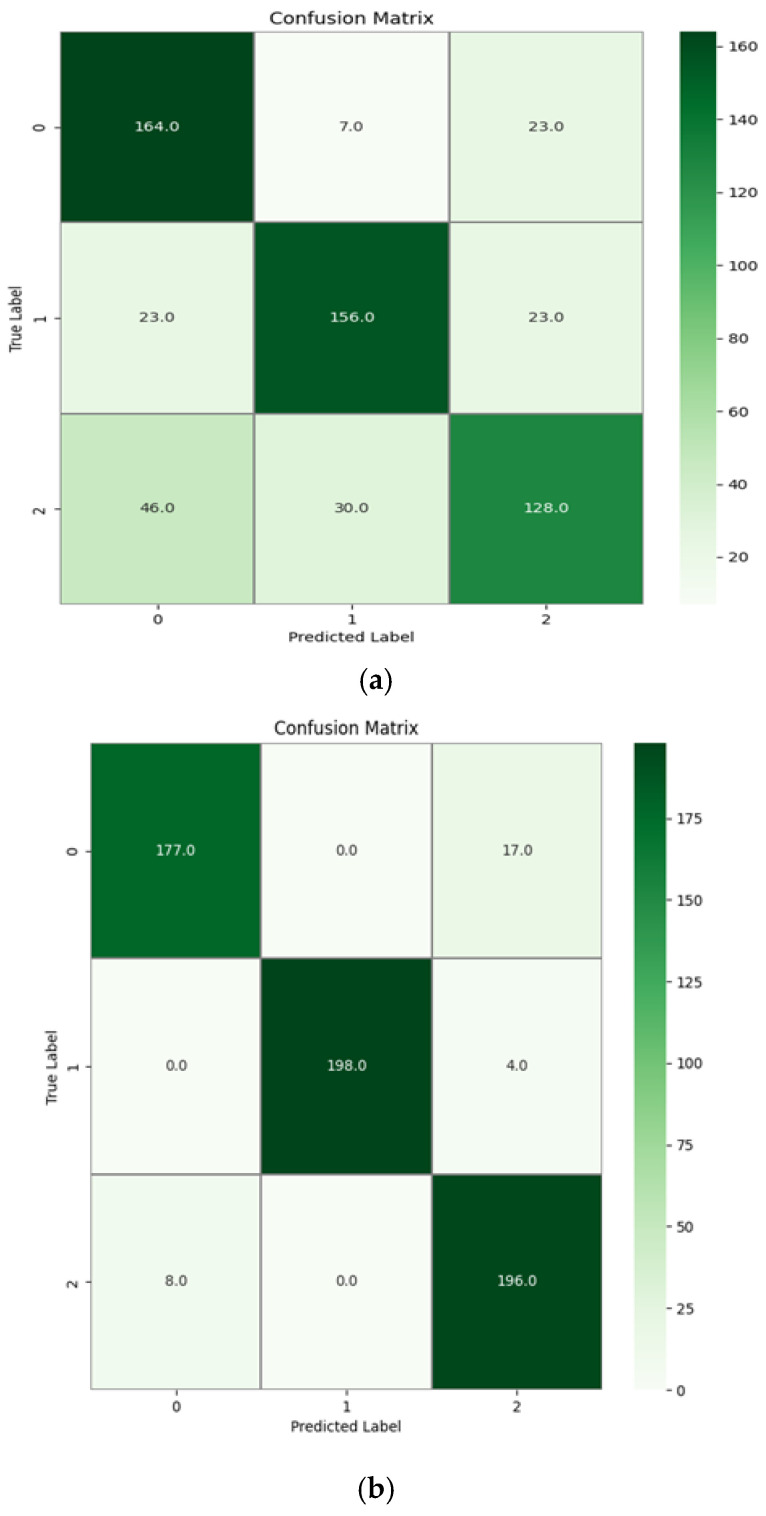
(**a**) The confusion matrix for a subject’s data without empirical error removal and (**b**) the confusion matrix for a subject’s data with empirical error removal. The confusion matrix represents the classification results for a single subject’s data, not an average across multiple subjects.

Figure 14a shows a graphical representation of the confusion matrix plotted for a subject’s data, which were given as test data to the trained model without the empirical error removal. The diagonal line represents the correctly predicted samples of the classification task for the MI-EEG data. Figure 14b shows the confusion matrix plotted for a subject’s data in which the error was removed using the empirical error model. It is clear from Figure 14b that the classification accuracy increased significantly after using the empirical error model. For the data without error removal, the misclassification rates are higher than that of the data after error removal. This proves that the proposed model was able to identify the artifacts that are caused due to motion and remove them from the data.

## 6. Conclusions

In this research study we presented the design and development of an empirical error model-based artifact removal method for the classification of MI-EEG data using a modified CNN-based deep learning algorithm for wheelchair users with severe mobility issues. The empirical error model has emerged from combination of the 8-channel MI-EEG data, the inertial sensor-based acceleration data of the subject in motion, the weight of the wheelchair, the weight of the subject, and the surface friction of the terrain of the wheelchair. Data were collected from five subjects. EEG data recordings were carried out in two different experimental setups. The first was the reference EEG data recording of a subject in an artifact-free setup. The second setup was the EEG data recording of the subject in motion with three different wheelchairs and five different surfaces. A total of seventy-five recordings were captured using the second experimental setup for the five subjects, which formed the dataset for the proposed study. To validate the empirical error model, performance metrics such as accuracy, precision, recall, and F1-score were measured before and after the application of the error model to the EEG data were measured for four different cases. It was observed that the performance metrics in the classification of the EEG data using the proposed LM-CNN method showed a maximum increase of 20% in accuracy, 18% in precision, 12% in recall, and 20% in F1-score when the empirical error model was applied to the EEG data compared to when the error model was not applied. While our model demonstrates success in EEG-based wheelchair navigation across five specific terrains, its broader applicability remains limited due to untested conditions and environments. Future research should focus on validating and expanding the model’s capabilities across diverse terrains, wheelchair configurations, and environmental conditions to enhance its practical real-world implementation and overall robustness.

## Figures and Tables

**Table 1:** Symbols used in the section.

Symbols	Definition
$E_{axs1}$	error calculated in x-axis for surface s1
$E_{axs2}$	error calculated in x-axis for surface s2
$E_{axs3}$	error calculated in x-axis for surface s3
$E_{axs4}$	error calculated in x-axis for surface s4
$E_{axs5}$	error calculated in x-axis for surface s5
$E_{aysn}$	error calculated in y-axis for a general surface
$E_{azsn}$	error calculated in z-axis for a general surface
${Cf}_{axs}$	correction factor with respect to the surface and error in x-axis
${Cf}_{ays}$	correction factor with respect to the surface and error in y-axis
${Cf}_{azs}$	correction factor with respect to the surface and error in z-axis
${µ}_{s1}$	Friction co-efficient (tire–s1)
${µ}_{s2}$	Friction co-efficient (tire–s2)
${µ}_{s3}$	Friction co-efficient (tire–s3)
${µ}_{s4}$	Friction co-efficient (tire–s4)
${µ}_{s5}$	Friction co-efficient (tire–s5)

**Table 2:** Symbols used in this section.

Symbols	Definition
$E_{axp1}$	error calculated in x-axis for subject weight w1
$E_{axpn}$	error calculated in x-axis for subject weight n
$E_{aypn}$	error calculated in y-axis for subject weight n
$E_{azpn}$	error calculated in z-axis for subject weight n
$W_{1}$	Weight of subject 1
$W_{2}$	Weight of subject 2
$W_{3}$	Weight of subject 3
$W_{4}$	Weight of subject 4
$W_{5}$	Weight of subject 5

**Table 3:** Symbols used in the section.

Symbols	Definition
$E_{axw1}$	error calculated in x-axis for wheelchair weight U1
$E_{axwn}$	error calculated in x-axis for wheelchair weight n
$E_{aywn}$	error calculated in y-axis for wheelchair weight n
$E_{azwn}$	error calculated in z-axis for wheelchair weight n
$U_{1}$	Wheelchair weight 1
$U_{2}$	Wheelchair weight 2
$U_{3}$	Wheelchair weight 3

**Table 4:** Details of the subjects who took part in the test.

Subject	Age	Weight (kg)
1	21	73.9 (W1)
2	21	81 (W2)
3	27	85 (W3)
4	25	82 (W4)
5	44	78.30 (W5)

**Table 5:** Details of the surfaces on which the tests were conducted.

Surface	Friction Co-Efficient (Tire–Surface) µ
Marble (s1)	21
Road (s2)	21
Brick (s3)	27
Carpet (s4)	25
Concrete (s5)	44

**Table 6:** LM-CNN model evaluation compared with other CNN models.

Algorithm and Method Employed	Accuracy	Precision	Recall	F1-Score
LM-CNN + Feature extraction	80.23	65.52	78.24	78.71
CNN2 [38] + Feature extraction	51.92	51.55	51.92	47.11
CNN3 [39] + Feature extraction	70	55.65	60.1	47.56
CNN4 [27] + Feature extraction	75.62	74.32	74.12	72.32
Multi-scale-CNN + Feature extraction [51]	93.74	94.15	91.22	92.66
1D-CNN [52]	99.38	99.46	99.21	99.33

**Table 7:** CNN comparison model features.

CNN Algorithm	Algorithm Details
LM-CNN	CONV2D–2 layers (Tanh) MaxPool2d–2 layers Dense fully connected layer–100 neurons (selu) Dense fully connected layer–3 neurons (softmax)
CNN2	CONV2D–2 layers (relu) MaxPool2d–2 layers Dense fully connected layer–100 neurons (selu) Dense fully connected layer–3 neurons (softmax)
CNN3	CONV2D–1 layer (relu) MaxPool2d–1 layer Dense fully connected layer–256 neurons (selu) Dense fully connected layer–3 neurons (softmax)
CNN4	CONV2D–2 layers (relu) MaxPool2d–1 layer Dense fully connected layer–256 neurons (selu) Dense fully connected layer–3 neurons (softmax)

**Table 8:** Empirical error model evaluation.

Algorithm and Pre-Processing Method Employed	Accuracy	Precision	Recall	F1-Score
LM-CNN + EM + Feature extraction	94.40	94.41	96.2	90.4
LM-CNN + AC + Feature extraction	86.00	82.6	81.9	83.2
LM-CNN + EMD + Feature extraction	84.32	84.5	83.2	84.1
LM-CNN + DWT + Feature extraction	70.00	56.80	80.00	65.28

**Table 9:** Overall architecture evaluation.

Algorithm and Pre-Processing Method Employed	Accuracy	Precision	Recall	F1-Score
LM-CNN +EM + Feature extraction	94.40	94.41	96.2	90.4
CNN3 + AC + Feature extraction	76.4	76.2	76.12	76.23
CNN4 + EMD + Feature extraction	80.32	80.5	80.2	79.1

## Data Availability

The datasets generated during and/or analyzed for the current study are available from the corresponding author on reasonable request.
